# HOW DOES EXECUTIVE FUNCTION IMPACT ADHERENCE AND COMPLIANCE TO NOVEL PHYSICAL THERAPY INTERVENTION AMONG VETERANS?

**DOI:** 10.1093/geroni/igad104.3397

**Published:** 2023-12-21

**Authors:** Elisa Ogawa, Rebekah Harris, Rachel Ward, Mary-Kate Palleschi, William Milberg, David Gagnon, Ildiko Halasz, Jonathan Bean

**Affiliations:** VA Boston Healthcare System, Boston, Massachusetts, United States; VA Boston Healthcare System, Boston, Massachusetts, United States; VA Boston Healthcare System, Boston, Massachusetts, United States; VA Boston Healthcare System, Boston, Massachusetts, United States; VA Boston Healthcare System, Boston, Massachusetts, United States; Boston University, Boston, Massachusetts, United States; VA Boston Healthcare System, Boston, Massachusetts, United States; VA Boston Healthcare System, Boston, Massachusetts, United States

## Abstract

Executive function problems may make it difficult for a person to adhere to and comply with Physical Therapy (PT) treatment. This study examined adherence and compliance to a novel PT intervention among Veterans with and without executive function deficits (EFD+/EFD-). This study was a preplanned secondary analysis of an ongoing Randomized Controlled Trial among middle to older Veterans (≥50yrs) with slow walking speed. Participants’ adherence (dropouts) and compliance (attendance, post-treatment exercise) to 8-week PT treatment were evaluated based on their baseline EFD status. Seventy-two participants were included in this study (mean age 72) and 22% (n=16) were EFD+. Veterans with EFD+ at baseline had worse mobility both objectively (Short Physical Performance Battery, 7.7 ± 1.9 vs. 9.5 ± 2.2, p< 0.01) and subjectively (Activity Measure for Post-Acute Care (AM-PAC), 57.6 ± 4.7 vs. 62.2 ± 5.7, p< 0.01). Dropout rates for EFD+ and EFD- were 44% and 25%, respectively. In the multivariable logistic regression, dropouts were associated with pain interference, depressive symptoms, mobility, and living alone. There were no differences in treatment attendance or post-treatment exercise compliance based on EFD status measured by number of attended sessions or number of post-treatment exercise days. In conclusion, EFD+ was associated with poor mobility and higher rates of dropping out. Screening for EFD is critical and may assist in predicting individuals who will need more support to enhance mobility.

